# siRNA-induced ABCE1 silencing inhibits proliferation and invasion of breast cancer cells

**DOI:** 10.3892/mmr.2014.2424

**Published:** 2014-07-28

**Authors:** BO HUANG, HONGLI ZHOU, XIANPING LANG, ZHILIANG LIU

**Affiliations:** 1 Department of Thoracic Surgery, The First Affiliated Hospital of Liaoning Medical University, Jinzhou, Liaoning 121000, P.R. China; 2 Department of Urology, The First Affiliated Hospital of Liaoning Medical University, Jinzhou, Liaoning 121000, P.R. China

**Keywords:** breast cancer, siRNA, ABCE1

## Abstract

Breast cancer is the most common type of cancer among females and the adenosine triphosphate (ATP) binding cassette E1 (ABCE1) gene is a member of the ATP-binding cassette (ABC) family. Studies in lung cancer have shown that overexpression of ABCE1 in tumor cells promotes growth and inhibits apoptosis. However, little is known about whether the ABCE1 gene is associated with breast cancer. In the present study, ABCE1 expression was assessed in breast cancer tissue and adjacent normal breast tissue using immunohistochemistry. Furthermore, small interfering (si)RNA targeting ABCE1 was constructed and transfected into MCF-7 human breast cancer cells to downregulate ABCE1 expression. The effect of ABCE1 knockdown on cell proliferation, invasion, apoptosis and gene expression was then assessed using MTT assay, Transwell migration assay, flow cytometry and western blot analysis, respectively. ABCE1 was observed to be overexpressed in breast cancer tissue compared with adjacent normal breast tissue. Furthermore, ABCE1-siRNA was found to inhibit proliferation and invasion in breast cancer cells, significantly induce breast cancer cell apoptosis (P<0.05) *in vitro* and increase the protein expression of RNase L. These findings showed that ABCE1 had an important role in proliferation, invasion and apoptosis in MCF-7 human breast cancer cells and that ABCE1 may inhibit intracellular RNase L activity, which inhibits the 2-5A/RNase L pathway, interfering with the biological characteristics of breast cancer cells.

## Introduction

Breast cancer is a type of malignant tumor derived from breast ductal epithelial cells. Recurrence and metastasis are the predominant causes of mortality in patients with breast cancer, with no satisfactory treatments available at present (1). The ABCE1 gene is a member of the ATP-binding cassette (ABC) family and encodes a protein which was originally identified based on its capacity to inhibit ribonucleic L (RNase L), an interferon-induced nuclease in mammalian cells (2,3). RNase L has roles in cell apoptosis and proliferation and is a candidate tumor suppressor protein. ABCE1 has previously been reported to be involved in the development of small cell lung cancer and lung adenocarcinoma (4–6). However, the role of ABCE1 in metastasis and proliferation in breast cancer has yet to be elucidated. In the present study, RNA interference (RNAi) was used to knock down ABCE1 expression in MCF-7 human breast cancer cells to investigate the role and mechanism of ABCE1 in breast cancer progression. Phenotypic changes induced by ABCE1 knockdown, including changes in ABCE1 and RNase L protein levels, as well as changes in proliferation, invasion and apoptosis were assessed in the transfected MCF-7 cells.

## Materials and methods

### Breast cancer samples

Breast cancer samples were obtained from 50 patients with breast cancer who underwent surgery at the First Affiliated Hospital of Liaoning Medical University (Jinzhou, China) between January 2009 and December 2012. Patients had received no chemotherapy or radiotherapy prior to their enrollment in the present study. The acquisition and analysis of the breast cancer samples was approved by the ethics committee of the First Affiliated Hospital of Liaoning Medical University and informed consent was obtained from all patients.

### Cell culture

MCF-7 human breast cancer cells were obtained from the Shanghai Institutes for Biological Sciences (Shanghai, China). Cells were cultured in RPMI-1640 containing 10% fetal bovine serum, 10 U/l penicillin G and 100 mg/l streptomycin at 37°C in a humidified atmosphere with 5% CO_2_.

### Immunohistochemistry (IHC)

IHC was performed to analyze ABCE1 expression in breast cancer tissue and normal adjacent breast tissue. In brief, tissues were fixed with 4% paraformaldehyde and paraffin-embedded using standard methods. IHC was performed using specific rabbit polyclonal anti-ABCE1 antibodies diluted 1:250 (Jingmei Biotech Corporation, Beijing, China) according to the manufacturer’s instructions. Brown cytoplasmic staining was defined as a positive signal. Scoring criteria were used as described previously (7). In brief, the scoring was as follows: 1, <10% staining; 2, 10–25% staining; 3, 26–75% staining; and 4, >75% staining. The staining intensity was graded as follows: 0, negative staining; 1, weak staining; 2, moderate staining; and 3, strong staining. The staining index was calculated by multiplying the staining proportion score by the staining intensity score. The calculated staining index was then defined using a simplified scoring system as follows: 0, index 0–1; 1, index 2–4; 2, index 6–8; and 3, index 9–12. Staining scores of at least one were classified as positive staining.

### Plasmid construction

Based on the ABCE1 cDNA sequence in GenBank (https://www.ncbi.nlm.nih.gov/genbank/), the Basic Local Alignment Search Tool (http://blast.ncbi.nlm.nih.gov/Blast.cgi) was used to design three pairs of oligonucleotide sequences which were synthesized by Takara Biotechnology (Dalian) Co., Ltd (Dalian, China). The oligonucleotide sequences used were as follows: Forward, 5′-GAG TAC GTT TCC TGT GAA GCC ACA GAT GGG GTA AAC GTA CTC GCT GTA GCT TTT TTG-3′ and reverse, 5′-AAT TCA AAA AAG CTA CAG CGA GTA CGT TTA CCC CAT CTG TGG CTT CAC AGG TAA ACG TAC TCG CTG TAG CG-3′ for si-1; forward, 5′-GAT CCG AGT ACG ATG ATC CTC CTG ACT GTG AAG CCA CAG ATG GGT CAG GAG GAT CAT CGT ACT CTT TTT TG-3′ and reverse, 5′-AAT TCA AAA AAG AGT ACG ATG ATC CTC CTG ACC CAT CTG TGG CTT CAC AGT CAG GAG GAT CAT CGT ACT CG-3′ for si-2; and forward, 5′-GAT CCG CGA GAC CTC AGT ATG TTA CCT GTG AAG CCA CAG ATG GGG TAA CAT ACT GAG GTC TCG CTT TTT TG-3′ for si-N (control group). The oligonucleotides were annealed and ligated using RNAi-Ready pRNAT-U6.1/Neo-siRNA and the T4 DNA ligase. The recombinant plasmids were termed ABCE1-small interfering (si)RNA-1, ABCE1-siRNA-2 and ABCE1-siRNA-N and were sequenced.

### siRNA transfection

MCF-7 cells were seeded at a density of 2×10^5^ cells/well in six-well plates. After 24 h of incubation, cells were transfected with ABCE1-siRNA-1, ABCE1-siRNA-2 and ABCE1-siRNA-N in serum-free medium using Lipofectamine™ 2000 (Invitrogen Life Technologies, Carlsbad, CA, USA). Vector (10 μg) and 20 μl Lipofectamine 2000 were mixed and incubated for 15 min at room temperature. The solution was then added to the MCF-7 cells and incubated without serum or antibiotics for 6 h, after which the medium was replaced with medium containing serum, but no antibiotics.

### Western blot analysis of ABCE1 and RNase L

Cells were washed three times with ice-cold phosphate-buffered saline (PBS) then lysed in buffer for 30 min on ice. Protein samples were electrophoresed using 10% SDS-PAGE and transferred to nitrocellulose membranes. Nonspecific reactivity was blocked using 5% nonfat dry milk in Tris-buffered saline containing Tween 20 for 1 h at room temperature. Membranes were then incubated with rabbit anti-human ABCE1 or RNase L antibodies diluted 1:250 (Jingmei Biotech Corporation) overnight at 4°C, followed by incubation with goat anti-rabbit antibodies diluted 1:500 (Jingmei Biotech Corporation). Membranes were also probed with β-actin antibodies (Sigma-Aldrich, St. Louis, MO, USA) as an internal control.

### Cell proliferation assay

Cells were treated with the aforementioned siRNA for 48 h. Cells were then seeded at a density of 2×10^3^/200 μl in 96-well microplates. After 24, 48 and 72 h of culture, 20 μl MTT solution diluted to a concentration of 5 mg/ml in PBS, was added and the cells were incubated at 37°C for 4 h. The supernatant was then removed and 150 μl dimethyl sulfoxide was added to each well. The dark-blue MTT crystals were dissolved by shaking the plates at room temperature for 10 min and the absorbance was read using a Bio-Rad microplate reader (Bio-Rad, Hercules, CA, USA) with a test wavelength of 490 nm and a reference wavelength of 570 nm.

### Fluorescence-activated cell sorting (FACS)

Cells were treated with the aforementioned siRNA for 48 h. Cells were then isolated and stained with phycoerythrin (PE) Annexin V and 7-aminoactinomycin D (7-AAD; BD Biosciences, Franklin Lakes, NJ, USA). Cells were analyzed using FACS in order to detect the fluorescence of PE Annexin V positive cells. The proportion of PE Annexin V positive cells in the siRNA-treated population was determined using the Super-Enhanced DMax method and the WinList software (Verity Software House Inc., Topsham, ME, USA).

### Invasion assays

Breast cancer cell invasion was assessed using Matrigel™-coated 24-well Transwell^®^ chambers (Corning Inc., Corning, NY, USA). Polycarbonate filters with a pore size of 8 μm, which separated the upper and lower compartments, were coated with 50 μg reconstituted basement membrane. At 48 h after transfection, serum-free RPMI-1640 containing 2×10^5^ cells in a volume of 300 μl was added to the upper compartment and the RPMI-1640 in the lower compartment was supplemented with 15% fetal calf serum. Cells in upper compartment were allowed to migrate to the lower compartment for 24 h at 37°C, after which the non-invasive cells on the upper surface of the membrane were removed using cotton swabs. The invasive cells attached to the lower membrane surface were fixed using 4% paraformaldehyde and stained with hematoxylin and eosin (H&E). The number of invasive cells was counted using a microscope (Olympus, Tokyo, Japan; magnification, ×200).

### Statistical analysis

Data were analyzed using SPSS 17.0 software (SPSS, Inc., Chicago, IL, USA). One-way analysis of variance was performed for multiple comparisons. P<0.05 was considered to indicate a statistically significant difference.

## Results

### ABCE1 expression in 50 patients with breast cancer

**Figure 1:**
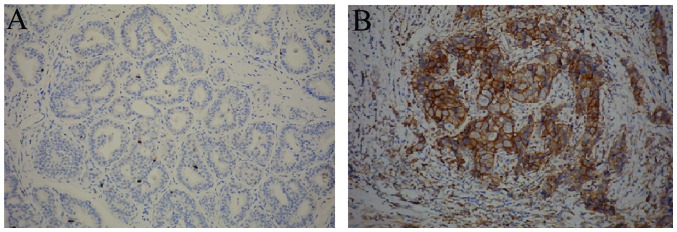
ABCE1 expression in a patient with breast cancer showing positive ABCE1 expression in the cytoplasm. ABCE1 expression in (A) healthy tissue adjacent to the breast cancer and (B) breast cancer tissue (magnification, ×200). ABC, ATP-binding cassette.

In order to investigate the role of ABCE1 in breast cancer, ABCE1 expression was analyzed in breast cancer tissue compared with adjacent healthy breast tissue. As shown in Fig. 1 and Table I, positive ABCE1 staining was predominantly observed in the cytoplasm. Furthermore, ABCE1 expression was found to be significantly higher in the breast cancer tissue compared with the adjacent healthy breast tissue (P<0.01), suggesting that ABCE1 may have a role in breast cancer.

### ABCE1-siRNA inhibits ABCE1 expression in breast cancer cells

**Figure 2:**
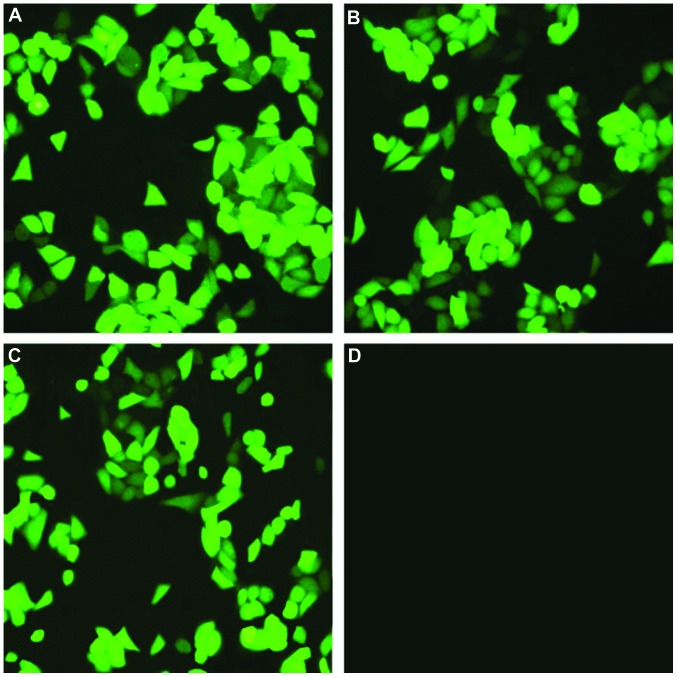
ABCE1-siRNA-induced changes in MCF-7 cell morphology 48 h after transfection. MCF-7 cells transfected with (A) ABCE1-siRNA-1 (B) ABCE1-SiRNA-2, (C) ABCE1-SiRNA-N and (D) untransfected MCF-7 cells. Magnification, ×100. ABC, ATP-binding cassette; si, small interfering.

In order to investigate the role of ABCE1 in breast cancer, siRNA knockdown of the ABCE1 gene was performed in the MCF-7 breast cancer cell line. ABCE1-RNAi was found to induce morphological changes in MCF-7 human breast cancer cells. Following transfection with ABCE1-siRNA-1 and ABCE1-siRNA-2, cytoplasmic green fluorescence was observed in the MCF-7 cells as ABCE1-siRNA carried green fluorescence. Furthermore, cells treated with ABCE1-siRNA were found to be less confluent or smaller and rounder compared with the control cells (Fig. 2). In accordance with this finding, fewer cells were observed upon treatment with ABCE1-siRNA-1 and ABCE1-siRNA-2 compared with ABCE1-SiRNA-N and in the control cells 72 h after transfection. The decrease in MCF-7 cell number induced by ABCE1-siRNA treatment suggests that ABCE1 has a role in cell cycle progression and survival in MCF-7 cells.

### Western blot analysis of ABCE1 and RNase L

**Figure 3:**
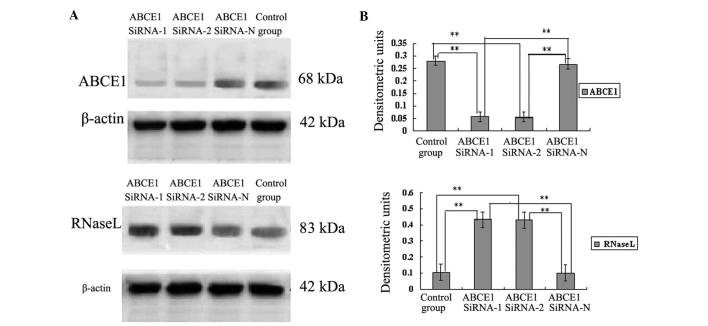
(A) Expression of ABCE1 and RNase L in MCF-7 human breast cancer cells detected using western blot analysis. Lane 1, MCF-7 cells transfected with ABCE1-siRNA-1; lane 2, MCF-7 cells transfected with ABCE1-siRNA-2; lane 3, MCF-7 cells transfected with ABCE1-siRNA-N; and lane 4: control, untransfected MCF-7 cells. (B) ABCE1 protein expression was significantly downregulated following transfection with ABCE1-siRNA-1 and ABCE1-siRNA-2 compared with the untransfected cells or those transfected with ABCE1-siRNA-N. RNase L protein expression was upregulated following transfection with ABCE1-siRNA-1 and ABCE1-siRNA-2 compared with the untransfected cells or those transfected with ABCE1-siRNA-N. ^**^P<0.05. ABC, ATP-binding cassette; si, small interfering.

In order to assess whether ABCE1-siRNA effectively silenced ABCE1 expression in MCF-7 cells, protein was extracted from the MCF-7 cells. Cell lysates were analyzed for ABCE1 and RNase L protein expression using western blot analysis with anti-ABCE1 or anti-R Nase L antibodies. As shown in Fig. 3, following transfection with ABCE1-siRNA-1 and ABCE1-siRNA-2, a significant decrease in ABCE1 protein expression and a significant increase of RNase L expression was observed. ABCE1 protein levels were found to be significantly downregulated, while RNase L levels were observed to be significantly upregulated following transfection with ABCE1-siRNA-1 and ABCE1-siRNA-2 compared with the untransfected cells or those transfected with ABCE1-siRNA-N.

### Cell proliferation assay

**Figure 4:**
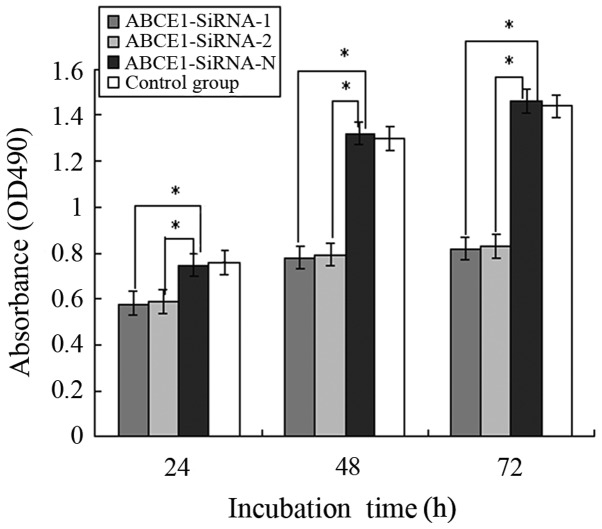
Decrease in MCF-7 cell proliferation following transfection with ABCE1-siRNA. MCF-7 human breast cancer cells were seeded on 96-well microplates, cultured for 24, 48 and 72 h and stained with MTT. The number of viable cells was determined using their absorbance. ^*^P<0.05. Data are representative of one of three independent experiments. ABC, ATP-binding cassette; si, small interfering; OD490, optical density at 490 nm.

MCF-7 cell proliferation was analyzed using the MTT assay. As shown in Fig. 4, compared with the MCF-7 cells transfected with ABCE1-siRNA-N, the proliferation of the MCF-7 cells transfected with ABCE1-siRNA-1 and ABCE1-siRNA-2 was significantly reduced to 77.33 and 78.66% (P<0.05), 59.09 and 59.85% (P<0.05) and 56.14 and 56.85% (P<0.01) at 24, 48 and 72 h, respectively. No significant difference was observed between the untransfected MCF-7 cells and those transfected with ABCE1-siRNA-N (P>0.05).

### Cell invasion assay

**Figure 5:**
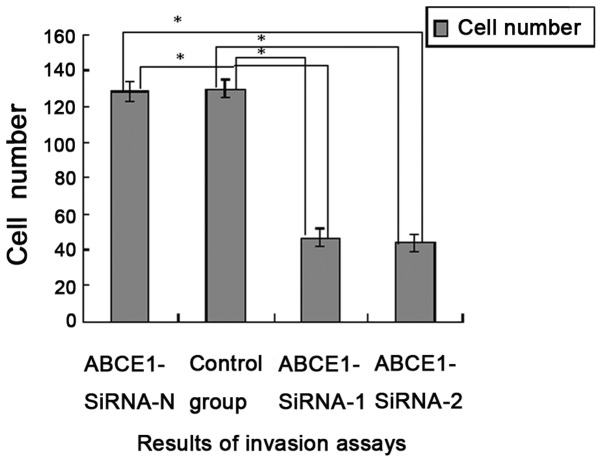
ABCE1-siRNA-1- and ABCE1-siRNA-2-transfection in MCF-7 cells reduced cell invasion in the Transwell assay as compared with untransfected MCF-7 cells or those transfected with ABCE1-siRNA-N. ^*^P<0.05. ABC, ATP-binding cassette; si, small interfering.

To investigate the role of ABCE1-siRNA in breast cancer cell invasion, Transwell invasion assays were performed. Untransfected MCF-7 cells and those transfected with ABCE1-siRNA-1, ABCE1-siRNA-2 and ABCE1-siRNA-N were incubated for 48 h on Matrigel-coated filters which were then H&E stained and analyzed using a microscope. The number of ABCE1-siRNA-1- and ABCE1-siRNA-2-transfected MCF-7 cells observed on the filter was significantly decreased compared with the number of untransfected and siRNA-N-transfected cells observed (P<0.05). Furthermore, no significant difference in cell number was found between the siRNA-N group and the control group. (P>0.05) (Fig. 5). These data suggested that knockdown of ABCE1 using transient transfection with ABCE1-siRNA may inhibit breast cancer cell invasion *in vitro*.

### ABCE1-RNAi induces apoptosis in MCF-7 cells

**Figure 6:**
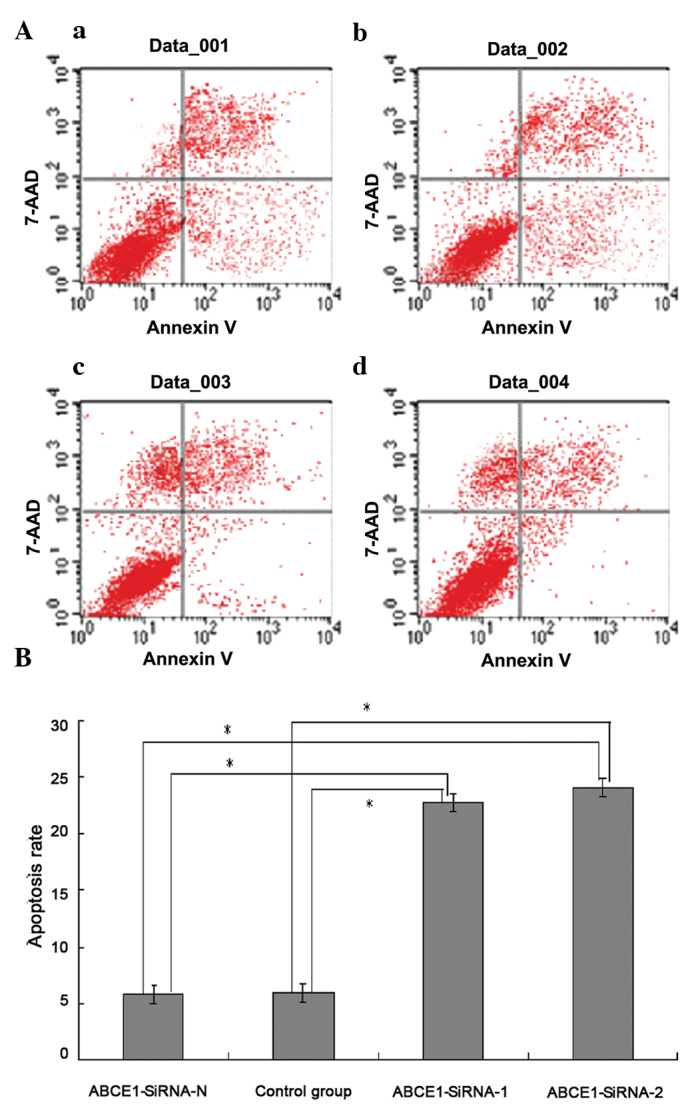
(A and B) ABCE1 knockdown in MCF-7 cells using ABCE1-siRNA-1 and ABCE1-siRNA-2 increases the rate of apoptosis compared with untransfected MCF-7 cells and those transfected with ABCE1-siRNA-N. Cells were collected and stained with PE Annexin V and 7-AAD. ^*^P<0.05. Dot plots are representative of one of three independent experiments. ABC, ATP-binding cassette; si, small interfering; PE, phycoerythrin; 7-AAD, 7-aminoactinomycin D.

The ABCE1-siRNA-induced decrease in MCF-7 cell number suggested that ABCE1-siRNA induced apoptosis in MCF-7 cells. To assess this hypothesis, PE Annexin V and 7-AAD staining and FACs analysis were performed, which showed that these cells underwent apoptosis (Fig. 6). These data suggested that ABCE1 regulated an anti-apoptotic pathway in MCF-7 cells and that the inactivation of ABCE1 led to the induction of apoptosis.

## Discussion

Breast cancer is one of the most common malignant tumor types in females. At present, breast cancer treatment options include, operation, chemotherapy and radiotherapy, as well as endocrine and biological treatment. However, the adverse reactions caused by operation, chemotherapy and radiotherapy have limited the development of traditional treatment (1). Therefore, the development of novel treatments for breast cancer has become an important focus for clinicians. Breast cancer development is a complex process and numerous genes are associated with the development and occurrence of breast cancer (8). The investigation of novel breast cancer-associated gene expression and elucidation of the role of such gene expression in breast cancer is important for the diagnosis, treatment and prevention of breast cancer.

ABCE1 is located on chromosome 4q31 in humans and the full-length cDNA encodes 599 amino acids with a molecular mass of 67,515 Da. As a member of the ABC superfamily, the ABCE1 protein possesses a conserved structure of the family and is located in the cytoplasm and mitochondria (9). ABCE1 has a unique N-terminal region containing eight conserved cysteine residues and is a highly conserved gene which is universally found in eukarya and archaea, but not in bacteria. ABCE1 is universally expressed in tissues, with high expression found in the brain, kidney and prostate and low expression in the lung, liver, spleen, heart and pancreas. No expression is found in the bone and spinal cord (10,11). In the present study, ABCE1 protein was observed to be significantly overexpressed in breast cancer tissue compared with adjacent normal breast tissue (P<0.01). This suggested that ABCE1 may be involved in the occurrence and development of breast cancer.

The ABCE1 protein was initially identified as an RNase L inhibitor, which specifically binds to RNase L and inhibits its activity. RNase L was first described as an 83,539 Da protein containing 741 amino acids. Structural analysis of RNase L has revealed that it consists of three domains: An N-terminal ankyrin repeat domain, a protein kinase homology domain and a C-terminal ribonuclease domain. Studies have shown that ABCE1 may inhibit RNase L activity, thereby blocking the 2-5A/RNase L pathway, which interferes with cell metabolism, inhibiting cell apoptosis (12,13). The ABCE1 gene has been reported to be overexpressed in numerous tumors, including colon, rectal, lung and small cell lung cancer (14–16). Furthermore, ABCE1 has been found to be involved in the occurrence and development of lung cancer, and RNase L has been shown to affect the pathogenesis of prostate cancer (16). As an RNase L inhibitor, ABCE1 may be implicated directly or indirectly in prostate cancer formation and/or metastasis (17). The present study identified that ABCE1 was highly expressed in breast cancer and that breast cancer malignancy was correlated with ABCE1 expression. These findings suggested that ABCE1 expression may be associated with breast cancer invasion and metastasis.

In the present study, a decrease in ABCE1 expression and an increase in RNase L expression was observed in breast cancer. When RNase L expression increased, the apoptotic rate significantly increased and cell proliferation and differentiation were reduced. This may be one of the mechanisms through which ABCE1 affects breast cancer proliferation, invasion and apoptosis.

The present study revealed that the treatment of human breast cancer cells with ABCE1-siRNA caused morphological and biochemical changes. Although ABCE1-siRNA was found to inhibit ABCE1 expression in MCF-7 cells, it had no significant effect on their viability. This finding suggested that ABCE1 may be an ideal target for cancer therapy. In conclusion, the present study provided an enhanced understanding of the physiological role of ABCE1 in breast cancer and may provide novel insight for the development of gene therapy technology to treat patients with breast cancer.

## Figures and Tables

**Table I:** Clinical characteristics and ABCE1 expression in patients with breast cancer.

		Staining score of ABCE1				
			
Variables	Patients (n)	0	1	2	3	P-value
Tissue						
Cancer	50	1	4	8	37	
Adjacent tissue	50	28	8	8	6	<0.001
Age (years)						
≤45	7	0	1	1	5	
>45	43	1	3	7	32	0.899
Tumor size						
≤2	10	0	2	3	5	
>2	40	1	2	5	32	0.171
Lymphnode metatasis						
Yes	30	0	2	5	23	
No	20	3	5	5	7	0.010
Histology grading						
I	16	3	4	2	7	
II	21	1	0	4	16	
III	13	0	1	0	12	0.013

ABC, ATP-binding cassette.
